# Evaluation of clinical outcomes of patients with post-stroke wrist and finger spasticity after ultrasonography-guided BTX-A injection and rehabilitation training

**DOI:** 10.3389/fnhum.2015.00485

**Published:** 2015-09-02

**Authors:** Li Jiang, Zu-Lin Dou, Qing Wang, Qiao-Yuan Wang, Meng Dai, Zhen Wang, Xiao-Mei Wei, Ying-Bei Chen

**Affiliations:** ^1^Department of Rehabilitation Medicine, The Third Affiliated Hospital, Sun Yat-sen UniversityGuangzhou, China; ^2^Institute of Medical Information, School of Biomedical Engineering, Southern Medical UniversityGuangzhou, China; ^3^Department of Ultrasound Medicine, The Third Affiliated Hospital, Sun Yat-sen UniversityGuangzhou, China; ^4^Robert D. and Patricia E. Kern Center for the Science of Health Care Delivery, Mayo ClinicRochester, MN, USA; ^5^Division of Health Care Policy and Research, Department of Health Sciences Research, Mayo ClinicRochester, MN, USA

**Keywords:** muscle spasticity, ultrasonography guidance, intramuscular injection, botulinum toxin type A, post-stroke rehabilitation

## Abstract

**Objective**: Using ultrasonography (US) to guide botulinum toxin type A (BTX-A) injection in patients with post-stroke wrist and finger flexor muscle spasticity and assessing clinical outcomes after the injection and rehabilitation intervention.

**Methods**: Twenty-three patients with wrist and finger spasticity after stroke were recruited in this study from May 2012 to May 2013. Under US guidance, the proper dose (250 U) of BTX-A was injected into each spastic muscle at two injection sites. Then, conventional rehabilitation training started next day after BTX-A injection. The degree of spasticity was assessed by modified Ashworth scale (MAS) and wrist and finger motor function by active rang of movement (AROM), and Fugl-Meyer assessment (FMA) at the baseline, 1, 2, 4 and 12 weeks after BTX-A injection.

**Results**: Significant decreases (*p* < 0.02) in the MAS scores of both the finger flexor muscle tone and wrist flexor muscle tone measured at 1, 2, 4, and 12 weeks after the BTX-A injection were found in comparison with the baseline scores. Compared with the baseline, the AROM values of the wrist and finger extensions and the FMA scores of the wrist and hand significantly increased (*p* < 0.02) at 2, 4 and 12 weeks after the BTX-A injection.

**Conclusions**: US-guided BTX-A injection combined with rehabilitation exercise decrease spasticity of the wrist and finger flexor muscles and improve their motor function in stroke patients up to 12 weeks following BTX-A injection.

## Introduction

Spasticity is a major cause of motor control deficits post stroke. Approximately one-third of stroke patients develop muscle spasticity and these patients often require specific treatment. It has been reported that upper extremity hypertonia (Ashworth score >1) occurs in 63% of patients with initial paralysis during the first 26 weeks post-stroke (Van Kuijk et al., 2007). Long-term spasticity can lead to a seriesof complications, including secondary muscle atrophy and joint deformity. Moreover, the spastic posture and deformity of the affected limbs often cause particular function impairment. For example, frequent flexor spasticity of the upper limb commonly impairs motor function of the hand, especially when the patient intends to open the hand and grasp an object. Patients with a functionless rigid hand have to face many daily living problems such as personal hygiene, eating and dressing. As a result, their quality of life is greatly decreased. One of major goals for post-stroke treatment is to reduce spasticity for improved motor function.

Botulinum toxin type A (BTX-A) causes a neuromuscular block in acetylcholine release and can prevent neuromuscular transmission and muscle contraction. BTX-A is regarded as an effective treatment agent, and the efficacy and safety of BTX-A injected in post-stroke patients with lower limb spasticity have been suggested in few limited-scale randomized-controlled trials (Mancini et al., 2005; Farina et al., 2008) and in a meta-analysis study (Rosales and Chua-Yap, 2008).

However, a successful and safe therapy with BTX-A requires anatomically accurate administration of BTX-A into the muscle belly, as it is well known that BTX-A can cause negative effects on the adjacent normal muscles. Knowing the location of the needle can help clinicians more accurately inject BTX-A into the target muscle. So far, manual needle placement (MNP), electromyography (EMG), electrical stimulation (ES), and ultrasonography (US; Schroeder et al., 2006) have all been applied to guiding BTX-A injection. According to the European consensus for the use of BTX-A in adult spasticity (Wissel et al., 2009), these techniques can aid muscle localization; however, the consensus does not provide recommendations of the most suitable techniques for aiding identification of target muscles for BTX-A injection. Furthermore, no detailed procedures for the accurate placement of the needle using US imaging has yet been provided. Therefore, additional studies are needed to clarify these issues.

US is a well-established reliable and reproducible imaging method for defining muscle anatomy. An ultrasound system with a 7.5 MHz linear transducer can provide sufficient resolution for both superficial and deep-seated muscles (Willenborg et al., 2002). As an alternative to electrophysiological techniques, US offers a visually controlled injection of BTX-A (Berweck et al., 2002, 2004). A study comparing MNP, ES, and US-guidance for BTX-A injection into the gastrocnemius of adults with spastic equinus after stroke indicated that the modified Ashworth scale (MAS) score was improved more in the US and ES groups than in the MNP group, while the ankle passive range of motion (PROM) had a greater increase in the US group than in the ES and MNP groups (Picelli et al., 2012). In China, physicians in Physical Medicine and Rehabilitation have limited training using electrophysiological techniques and thus do not widely apply ES guidance to administration of BTX-A injection.

Given that accurately injecting BTX-A into the right location of the target muscle is vitally important for effectively treating spasticity with this approach, the current study aimed at using sonographic imaging to guide BTX-A injection in patients with post-stroke wrist and finger spasticity and to assess the follow-up motor function outcomes. The results of the study provide useful information related to helping make accurate focal BTX-A injection in clinical practice.

## Materials and Methods

### Subjects

Post-stroke patients treated in the Department of Rehabilitation Medicine, The Third Affiliated Hospital (TAH), Sun Yat-sen University were recruited from May 2012 to May 2013. All participants were informed regarding the aims and contents of the study and provided written consent to participate. The study was approved by the Institutional Ethics Committee of the TAH. The inclusion criteria were as follows: age >18 years; arm spasticity as a consequence of ischemic or hemorrhagic stroke, diagnosed by computed tomography scan or magnetic resonance imaging; wrist and finger flexors tone graded between 1+ and 3 on the MAS; and time from stroke onset at least 3 months. The exclusion criteria were as follows: time from stroke onset over 1 year; contracture deformity in the upper limbs; infection at the injection site; oral medication use such as aminoglycoside antibiotics that can disturb the transmission of chemicals in the neuro-muscular junction; unstable medical condition; and severe cognitive disorders, diplegia, pregnancy, breast-feeding, history of BTX-A treatment, and other neurological diseases.

### Equipment and Medication

In this study, the injection of the needle into the target muscle was visually guided using an ultrasound device (Voluson 730 Expert; GE Medical Systems Kretztechnik GmbH and Co. OHG, Zipf, Austria). The frequency range of the GE M12L linear array ultrasound probe was 9.0–13.0 MHz.

BTX-A (BOTOX, 100 units/vial; Allergen Inc., Irvine, CA, USA) was diluted with 2 ml of 0.9% sodium chloride solution.

### Ultrasound-Guided BTX-A Injection

#### Target Muscles and Corresponding Dosage of BTX-A

Five muscles, including the flexor carpi radialis (FCR), flexor carpi ulnaris (FCU), flexor digitorum profundus (FDP), flexor digitorum superficialis (FDS), and pronator teres (PT), were selected as the target muscles in this study. The dosage of BTX-A for each target muscle was determined according to the mass of the target muscle and its spastic severity. Although the BTX-A dosage differed from muscle to muscle in a given patient, the total dosage (i.e., 250 U) used for the five muscles in each patient was the same among all the patients.

#### Sonographic Imaging Guidance

**Figure 1:**
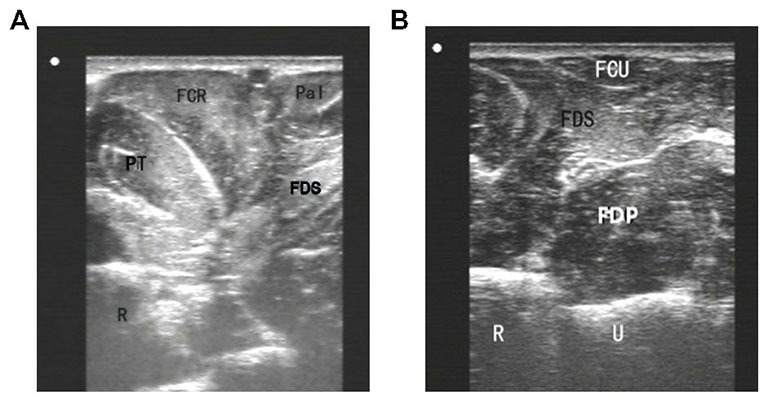
**Sonographic imaging of forearm muscles. (A)** an image obtained by the probe located at upper third of forearm anterior; **(B)** an image obtained by the probe located at upper third of forearm medialis. FCR, flexor carpi radialis; FCU, flexor carpi ulnaris; FDS, flexor digitorum superficialis; FDP, flexor digitorum profundus; Pal, rum superficialis; PT, pronator teres; R, radial; U, ulna.

**Figure 2:**
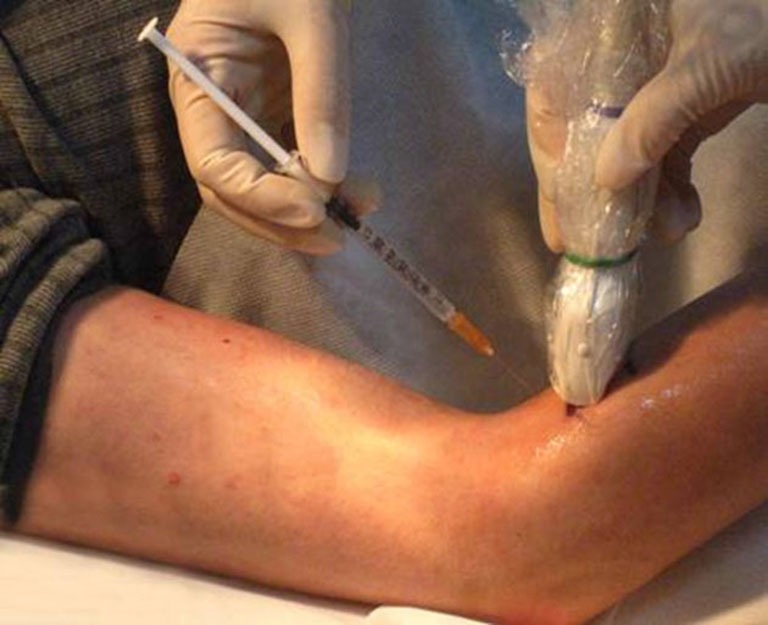
**Sonography guidance of BTA-X injection into FCR**. The angle between the needle and the skin was approximately 30°. The probe was placed on the target muscles at the skin surface site.

**Figure 3:**
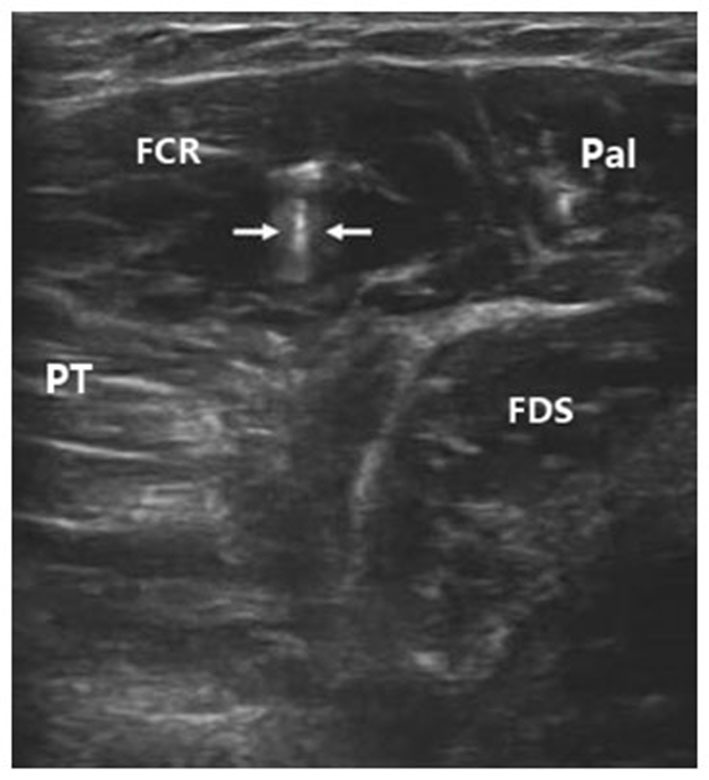
**Sonographic image indicating the inserted needle (hyperecho between two arrows) in FCR**. FCR, flexor carpi radialis; FDS, flexor digitorum superficialis; Pal, palmaris longus; PT, pronator teres.

To inject BTX-A into the FCR muscle, sonographic imaging was performed to guide the injection. The steps of the procedure were as follows: (1) the patient was placed in the supine position with the elbow extended and the forearm supinated (palm facing up). Patients who could not make or maintain the above positions were provided with proper assistance to achieve the needed body and arm postures; (2) the skin location for injection into the FCR muscle was identified by using the fingerbreadth measurement methods of Delagi et al. (2005) and was subsequently marked; (3) the transducer was located at the marker and positioned perpendicularly to the forearm surface to obtain a transverse view of the FCR. By adjusting the parameters such as view depth, focus, and gain, a clear image of the FCR muscle and other muscles could be displayed (Figure 1). In the transverse (axial) view, the target muscle was scanned from proximal to distal direction or* vice versa* till the largest cross-sectional area was identified; (4) To confirm the surface injection site, in some cases, the target muscle was stretched passively to visualize the dynamic contracture of the muscle; and (5) For optimal visualization of the needle point throughout the injection, the angle between the needle and the skin was kept approximately 30° (Figure 2; Picelli et al., 2014). The position of the inserted needle was shown on real-time sonographic images (Figure 3). Pre-injection sonographic images and the images during the injection were collected and stored for record. The second injection site of each target muscle was identified by distally moving the transducer approximately 1.5–2.0 cm. BTX-A was injected in the FCR at 2 sites, with 25 U for each site.

Similar injection procedures were followed for the PT, FCU, FDP, and FDS muscles. All the BTX-A injections were performed by the same doctor with prior experience in using US-guidance for the injection.

### Clinical Outcome Assessments for Motor Function

The clinical outcomes of the patients’ motor function were evaluated before the treatment of BTX-A (baseline), and 1, 2, 4 and 12 weeks after the injection by the same therapist. Three clinical outcome measures were assessed and the procedures were summarized below.

#### Modified Ashworth Scale (MAS)

The MAS scores were recorded to evaluate the degree of spasticity. The evaluation standards refer to a grading system scoring on a scale of 0–4 (Bohannon and Smith, 1987), where 0 is no increase in muscle tone; 1 is a slight increase in muscle tone at the end of the range of motion; 1+ is a slight increase in muscle tone through less than half of the range of motion; 2 is a more marked increase in muscle tone through most of the range of motion; 3 is a considerable increase in muscle tone; and 4 means a rigid joint.

For statistical purposes, a score of 1 was considered as 1, 1+ as 2, and scores of 2, 3 and 4 were recorded as 3, 4 and 5, respectively.

#### Active Range of Movement (AROM)

The AROMs of the wrist and finger joints were measured using a goniometer. To measure the AROM of the wrist, the patients were instructed by a therapist to perform an active wrist extension as far as possible from a flexed wrist position till the active force produced by the extensors (agonists) was balanced by the passive resistance from the stretched structures together with the spastic co-contraction in the flexors (antagonists). The AROM of the fingers was evaluated in a similar manner.

#### Fugl-Meyer Assessment (FMA) Scale

The FMA scale is widely accepted as a feasible and appropriate method of assessment of motor recovery in stroke rehabilitation. The simple FMA scale was used in this study to evaluate motor function of the wrist and hand. Herein, 33 items were included in the evaluation of the wrist and hand motor function. Each item was scored on a scale of 0–2, with higher scores indicating better motor function (Gladstone et al., 2002). The total score ranged from 0 (hemiplegia) to 66 points (normal motor performance). The aforementioned three assessments were performed by the same therapist who did not know whether the patients were injected with BTX-A or not.

### Rehabilitation Training

**Figure 4:**
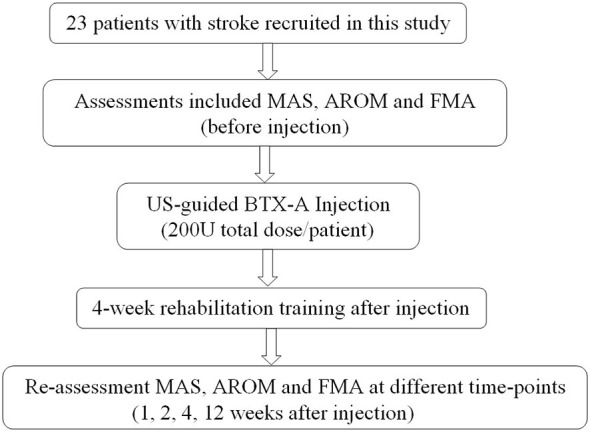
**Flow diagram of the experiment**. MAS, modified Ashworth scale; AROM, active range of motion; FMA, Fugl-Meyer assessment scale; BTX-A, botulinum toxin type A.

Conventional rehabilitation training was performed 1 day after the BTX-A injection, including joint traction, functional ES, and active motion. Upper limb orthotics were applied to maintain the patients’ normal joint movement for training if their condition did not allow performance of training activities without the orthotic assistance. The training lasted for 4 weeks with five sessions per week and 50 min for each session. After discharge from the hospital, the patients were asked to perform the rehabilitation training at home and were followed up for 12 weeks after the injection. Home training was carried out three sessions each week and 50 min each session for a total of 8 weeks. Family members were trained by a therapist to conduct the home training with the patients. Home training activities were recorded by the family members and were verified by a therapist weekly. Re-assessment of MAS, AROM and FMA at 1, 2, 4, 12 weeks after BTX-A injection was performed. Figure 4 shows the flow diagram of the experiment in this study.

### Statistical Analysis

We conducted descriptive analyses to summarize patients’ baseline characteristics (age, gender and time from stroke onset). All measurement data are presented as mean ± standard deviation. The pre-treatment and follow-up clinical evaluation scores were analyzed by nonparametric Wilcoxon matched-pairs signed-ranks tests without assumptions on normality of the data. Multiple comparisons (20 times) were treated with Bonferroni Correction and the significance level was set at *p* ≤ 0.05. Statistical analysis was conducted using Stata version 14 (StataCorp LP, College Station, TX, USA).

## Results

Twenty three subjects (13 males and 10 females) with wrist and finger spasticity were recruited in this study. The mean age of the patients was 58.2 ± 10.2 years (range, 35–70 years). The mean duration from disease onset was 7.4 ± 2.5 months (range, 3–12 months). Fifteen and eight cases had cerebral infarction and cerebral hemorrhage, respectively. All patients completed the requested assessments at all five time-points (baseline and 1, 2, 4 and 12 weeks after the injection). No apparent adverse effects were observed during the follow-up times.

### MAS Assessment

All patients showed an improvement in muscle tone according to the MAS data of the wrist and finger flexor muscles (Table 1). Significant decreases in both the flexor digitorum muscle tone and flexor carpi muscle tone measured at 1, 2, 4 and 12 weeks after the BTX-A injection were found in comparison with the baseline scores (*p* < 0.02). The MAS of wrist flexor muscle tone was lowest at 2 weeks during follow-up. The MAS scores of the wrist flexor muscle tone measured at 4 and 12 weeks tended to be slightly increased in comparison with the score measured at 2 weeks. Different from the MAS scores of wrist flexor muscles, the MAS score of the finger flexor muscle tone measured at 4 weeks was lowest compared with the MAS scores measured at other time-points (Table 1).

**Table 1:** **Changes in the MAS, AROM, and FMA scores measured before injection and at 1, 2, 4, 12 weeks after BTX-A injection (mean ± standard deviation)**.

		After injection (*n* = 23)Items			
Items	Before injection (*n* = 23)	Week 1	Week 2	Week 4	Week 12
MAS (score)
Wrist flexor	3.0 ± 0.6	2.2 ± 0.4 (*p* < 0.02*)	1.1 ± 0.3 (*p* < 0.02*)	1.3 ± 0.5 (*p* < 0.02*)	1.4 ± 0.5 (*p* < 0.02*)
Fingers flexor	3.1 ± 0.3	2.4 ± 0.6 (*p* < 0.02*)	1.4 ± 0.5 (*p* < 0.02*)	1.2 ± 0.4 (*p* < 0.02*)	1.3 ± 0.6 (*p* < 0.02*)
AROM (degree)
Wrist extensor	2.5 ± 1.9	2.8 ± 1.9 (*p* = 0.29)	7.0 ± 1.7 (*p* < 0.02*)	11.0 ± 1.9 (*p* < 0.02*)	16.0 ± 2.6 (*p* < 0.02*)
Fingers extensor	3.1 ± 2.1	3.5 ± 2.0 (*p* = 0.54)	6.6 ± 2.3 (*p* = 0.002*)	9.8 ± 2.5 (*p* < 0.02*)	13.0 ± 2.9 (*p* < 0.02*)
FMA (score)
Wrist-hand	8.8 ± 2.0	9.3 ± 2.1 (*p* = 0.94)	10.3 ± 2.5 (*p* = 0.002*)	13.1 ± 2.4 (*p* < 0.02*)	15.4 ± 2.7 (*p* < 0.02*)

Abbreviations: MAS, modified Ashworth scale; AROM, active range of motion; FMA, Fugl-Meyer assessment scale; BTX-A, botulinum toxin type A. All p values were adjusted with Bonferroni Correction (*20). *Statistically significant difference (p ≤ 0.05) in comparison with before injection.

### AROM Assessment

Although no significant improvement was found in the AROM for wrist extension at 1 week after the injection (*p* = 0.29), the AROM values were significantly increased at 2, 4, and 12 weeks (7.0 ± 1.7°, 11.0 ± 1.9°, 16.0 ± 2.6°, respectively; *p* < 0.02) in comparison with the baseline (2.5 ± 1.9°; Table 1). Similar significant increases in the AROM values of finger extension were also observed (AROM values at baseline, 2, 4, and 12 weeks: 3.1 ± 2.1°, 6.6 ± 2.3°, 9.8 ± 2.5°, 13.0 ± 2.9°; *p* < 0.02). The AROM results of both the wrist and fingers showed significant improvements (*p* < 0.02) from 2 to 12 weeks after the BTX-A injection.

### FMA Assessment

Compared with the baseline FMA results of the wrists and hands (8.8 ± 2.0), the FMA scores at 2, 4 and 12 weeks after the injection were significantly improved (10.3 ± 2.5, 13.1 ± 2.4, and 15.4 ± 2.7, respectively; *p* < 0.02), whereas the FMA score at 1 week (9.3 ± 2.1) remained unchanged from the baseline (*p* = 0.94; Table 1). Similar to the results of the AROM assessment, the FMA scores that present motor function of the wrist and hand maintained significant improvements from 2 weeks to 12 weeks after the BTX-A injection (*p* < 0.02).

## Discussion

BTX-A as a chemodenervating drug with reversible clinical effects, offers the possibility to paralyze muscles that contribute to spastic deformities. The BTX-A dose and correct identification of the spastic muscles are important factors influencing the treatment outcome. Chin et al. (2005) injected BTX-A with the aid of palpation of the target muscles to reduce limb spasticity in 226 children with cerebral palsy. They concluded that the accuracy of MNP was acceptable only for the gastrocnemius and soleus muscles (75%), whereas it was unacceptable for the forearm and hand muscles (13–35%). Schroeder et al. (2006) reviewed different injection techniques of BTX-A and suggested that US-guidance could be a reliable localization technique, by which the typical patterns of the target muscle could be recognized in a short period of time without causing pain. Therefore, sonographic imaging may potentially be a useful approach for guiding BTX-A injection into correction locations/muscles for treating adult patients with spasticity.

US guidance allows identification of the target muscles and verification of the position of the needle tip in the designated muscle mass. In addition, US helps identify blood vessels and nerves, and may help guide the needle to stay away from those structures. It has been suggested that US could help avoid BTX-A administration into the blood vessels and nerves, owing to improved injection accuracy (Henzel et al., 2010). There is little doubt that using US can help make BTX-A injections safer and more accurate.

The current study performed US-guided BTX-A injection to treat wrist and finger spasticity in post stroke patients in conjunction with rehabilitation intervention. Our target muscles for the injection included the FDP muscle of the forearm and due to the depth of this muscle, it is often difficult to determine the injection spot and depth. With the use of US guidance, the FDP was quickly identified and the injecting needle was inserted to the correct location within the muscle relatively easily. This outcome suggests that deep, small, or superficial muscles can all be clearly displayed upon the sonographic images to guide the injection. Without the guidance, it would be challenging to accurately deposit the drug in the deep musculature.

US-guided BTX-A injection is useful for decreasing spastic muscle tone in stroke patients. A previous study (Picelli et al., 2012) concluded that the US-guided injection into the gastrocnemius muscle could improve functional clinical outcomes in adults with spastic equinus after stroke. In another study by the same research group (Picelli et al., 2014), the authors demonstrated that the MAS scores of the wrist and fingers improved 4 weeks after US-guided injection in patients post stroke.

The MAS is often used to assess muscle spasticity level in clinical studies. Higher scores indicate more severe spasticity and lower scores mean less spasticity. In addition, the AROM, PROM and FMA represent common assessment items used for evaluating active movement and functional status. In the study by Picelli et al. (2014) on upper limb spasticity, only the MAS and PROM were used to assess the therapy outcomes after US-guided BTX-A injection but no information was provided regarding the active and functional activities of the patients. In our study, the AROM and FMA were included in the outcome assessments, in addition to the MAS assessment of spasticity, thereby allowing the improvement in patients’ active motor activities to be assessed after BTX-A injection. Indeed, the results from this study showed that the MAS, AROM, and FMA measures all improved in the patients up to 12 weeks after US-guided BTX-A injection. As opposed to the AROM and FMA data that did not improve until 2 weeks after the injection, the MAS scores of the wrist and finger flexors were obviously decreased only 1 week after the injection. In other words, the earliest benefit after US-guided BTX-A injection was seen on the muscle tone. The MAS score of the wrist flexors reduced further at 2 weeks, but increased slightly at 4 and 12 weeks compared to the value at 2 weeks. Similarly, the MAS score of the finger flexors decreased at each assessment after injection with the largest change in the observed at 4 weeks. The MAS measure indicated that reduced spasticity was maintained at 12 weeks after US-guided BTX-A injection. Moreover, both the AROM of the wrist and finger extensors and the FMA scores improved 2 weeks after US-guided BTX-A injection and the improvement was maintained throughout the rest of the 12 weeks follow-up.

BTX-A is effective for decreasing muscle spasticity. Rehabilitation exercises after the injection are essential for motor function improvement. Henzel et al. (2010) performed a double-blind randomized trial aimed at investigating the effects of treating the upper limbs spasticity in 24 post-stroke patients using BTX-A injection with and without ES therapy. The authors found that combined with ES therapy, the BTX-A injection induced significant improvements in the spastic muscle tone, movement range of the joint and daily living activities of the patients. The wrist and finger flexor muscles could be stretched more easily with an increase in AROM. Through comprehensive conventional rehabilitation exercises after the injection, including strengthening exercises of the agonistic and antagonistic muscles, coordination exercises of multiple joints, and functional ES of target muscles, spasticity and motor function can be more remarkably improved than BTX-A injection not accompanied rehabilitative exercises. Moreover, in the study led by Woldag and Hummelsheim (2003), the authors found that the AROM increased and MAS decreased significantly in the wrist flexors at 4, 8 and 12 weeks after BTX-A injection in stroke patients. Accordingly, we suggest that US-guided BTX-A injection combined with rehabilitation exercises can lead to functional benefits for stroke patient with wrist and finger flexor spasticity but BTX-A injection alone may not achieve such beneficial results.

It is well known that the effect of BTX-A injection varies over time. In the study by Picelli et al. (2014) on upper limb spasticity, clinical outcomes were only assessed at one time-point (4 weeks) after the injection, and therefore, the effect of time on the outcomes could not be determined. In this study, the follow-up time points were 1, 2, 4 and 12 weeks after injections and the results showed that the wrist and finger flexor spasticity of the patients subsided only 1 week after US-guided injection. At the other follow-up time points (2, 4 and 12 weeks), the changes were also significant but varied in amplitude. A previous study found that decreased MAS scores of the wrist and fingers were obvious 4 weeks after BTX-A injection (Woldag and Hummelsheim, 2003), which is similar to the outcome found in our study. The patients in that study were assessed at 4, 8 and 12 weeks after BTX-A injection, and the authors reported that the MAS, PROM, and AROM scores were all improved during the follow-up (Woldag and Hummelsheim, 2003). In the present study, similar results were observed in terms of the MAS and AROM measures of the wrist and fingers. To compare with previous studies (Woldag and Hummelsheim, 2003; Picelli et al., 2014) and to allow for a complete observation of spasticity therapy outcomes, we observed the outcomes not only in longer terms (4–12 weeks), but also in shorter terms (1–2 weeks). We consider that data observed at more time points are helpful to understand the spasticity tendency after BTX-A injection and to potentially create a proper therapy plan for patients with spasticity. However, it is still unclear whether the spasticity decreases more quickly by US-guided injection compared with other guidance methods.

Finally, this study used sonographic imaging online to guide BTX-A injection to treat post-stroke spasticity and satisfying outcomes were achieved. The US guidance provides real-time visual images of the needle placement and anatomic information of the target muscles. US-guided BTX-A injection combined rehabilitation exercises help decrease muscle spasticity and further improve upper limb motor function at short and longer follow-up time points. By demonstrating favorable results of combining US-guided BTX-A injection with conventional rehabilitation interventions with clinical outcome measures assessed at multiple follow-up time points, the current study provides new information relevant to efficacies of US-guided BTX-A injection and rehabilitation on treating muscle spasticity and improving muscular function.

This study, however, has limitations that include primarily the lack of a control group with MNP injection without US guidance. Very few patients expressed interest in MNP, and few patients wanted to accept US-guided injection without rehabilitation intervention in our hospital. These conditions made it difficult to establish a MNP + rehabilitation group and/or US-guided BTX-A injection without rehabilitation group. However, even without a control group we believe our results are valid based on findings reported by previous studies that have shown US-guided injection was more effective in improving outcome measures than MNP injection in stroke patients with lower and upper limb muscle spasticity (Picelli et al., 2012, 2014). Hesse et al. (1998) compared the clinical outcomes between interventions of BTX injection + ES and BTX injection alone in stroke patients with spasticity. The BTX + ES group had more spasticity reduction and better limb positioning at rest. Other research groups demonstrated BTX-A injection combining rehabilitation improved functional activities and decreased muscle spasticity in a small number of stroke survivors with wrist and finger spasticity (Woldag and Hummelsheim, 2003; Cardoso et al., 2007). In future studies on using US-guided BTX-A injection for treating spasticity of both upper and lower limbs in post-stroke patients, we would recruit more subjects by cooperation with other health care centers and launch more comprehensive clinical studies on whether US-guided injection has a better treatment result than other injection techniques and whether US-guided injection combined with a rehabilitation program is more effective in improving spasticity and motor function than the US-guided injection alone. We would also like to determine if spastic muscles at certain joints are more suitable for US-guided injection than others.

In conclusion, the results of this study suggest that the use of US can help accurately locate the target muscle and region within the muscle for needle placement, and that US-guided BTX-A injection combined with rehabilitation exercises can not only decrease spasticity of the wrist and finger flexor muscles at various times following the injection and rehabilitation, but can the approach also significantly improve motor function of the wrist and fingers in patients during post-stroke rehabilitation.

## Conflict of Interest Statement

The authors declare that the research was conducted in the absence of any commercial or financial relationships that could be construed as a potential conflict of interest.
